# Outer membrane protein folding from an energy landscape perspective

**DOI:** 10.1186/s12915-017-0464-5

**Published:** 2017-12-21

**Authors:** Bob Schiffrin, David J. Brockwell, Sheena E. Radford

**Affiliations:** Astbury Centre for Structural Molecular Biology, School of Molecular and Cellular Biology, Faculty of Biological Sciences, University of Leeds, Leeds, LS2 9JT UK

## Abstract

The cell envelope is essential for the survival of Gram-negative bacteria. This specialised membrane is densely packed with outer membrane proteins (OMPs), which perform a variety of functions. How OMPs fold into this crowded environment remains an open question. Here, we review current knowledge about OMP folding mechanisms in vitro and discuss how the need to fold to a stable native state has shaped their folding energy landscapes. We also highlight the role of chaperones and the β-barrel assembly machinery (BAM) in assisting OMP folding in vivo and discuss proposed mechanisms by which this fascinating machinery may catalyse OMP folding.

## The outer membrane protein folding problem

More than 50 years of work on the folding pathways of water soluble proteins has yielded a plethora of detailed insights into the conformations visited by polypeptides along complex routes to their native conformations. These include the structural and energetic properties of transition states [1–4], partially folded intermediates [5–7], and lowly-populated ‘invisible’ states [8]. The role of molecular chaperones in assisting folding and preventing aggregation has also been studied extensively [9, 10], and powerful biophysical and structural methods are beginning to reveal how water soluble proteins fold in the crowded cellular environment [11], including during their synthesis on actively translating ribosomes [12–14]. By contrast, understanding the folding energy landscapes of membrane proteins (MPs) has lagged behind those of water soluble proteins, despite recent important progress. Here, we focus on outer membrane proteins (OMPs) from Gram-negative bacteria (for discussions of α-helical MPs see [15–22], and elsewhere in this issue). OMP assembly in vivo is complicated by the requirement to fold in an asymmetric lipid bilayer, as well as the need to cross the inner membrane and periplasmic space following synthesis in the cytosol. We highlight how these requirements impose constraints on the evolution of OMP sequences and how this influences the thermodynamics and kinetics of OMP folding. Finally, we discuss how cellular proteins may sculpt the folding energy landscape of OMPs to increase the rate and/or efficiency of folding and assembly in vivo.

## Evolutionary constraints on OMP sequences

For proteins to fold stably into the hydrophobic environment of a biological membrane their structure must fulfil a number of energetic requirements including: (1) the hydrogen bonding potential of their polar backbone carbonyl and NH groups must be mostly satisfied [23] to offset the energetic cost of peptide bond burial (~ 1.2 kcal/mol [24]); and (2) the amino acid side chain groups in contact with the acyl chains of the lipid bilayer must be predominantly hydrophobic. The secondary structures of α-helical and β-barrel membrane proteins allow them to meet these requirements in different ways [25, 26]. The residues within each helix in α-helical MPs make backbone hydrogen bonds, allowing the separate insertion of helices into the membrane bilayer which can subsequently associate laterally to form their final native structure [27]. By contrast, β-barrel OMPs form cylindrical structures by making hydrogen bonds between residues in different β-strands, potentially far from each other in sequence [28].

**Fig. 1:**
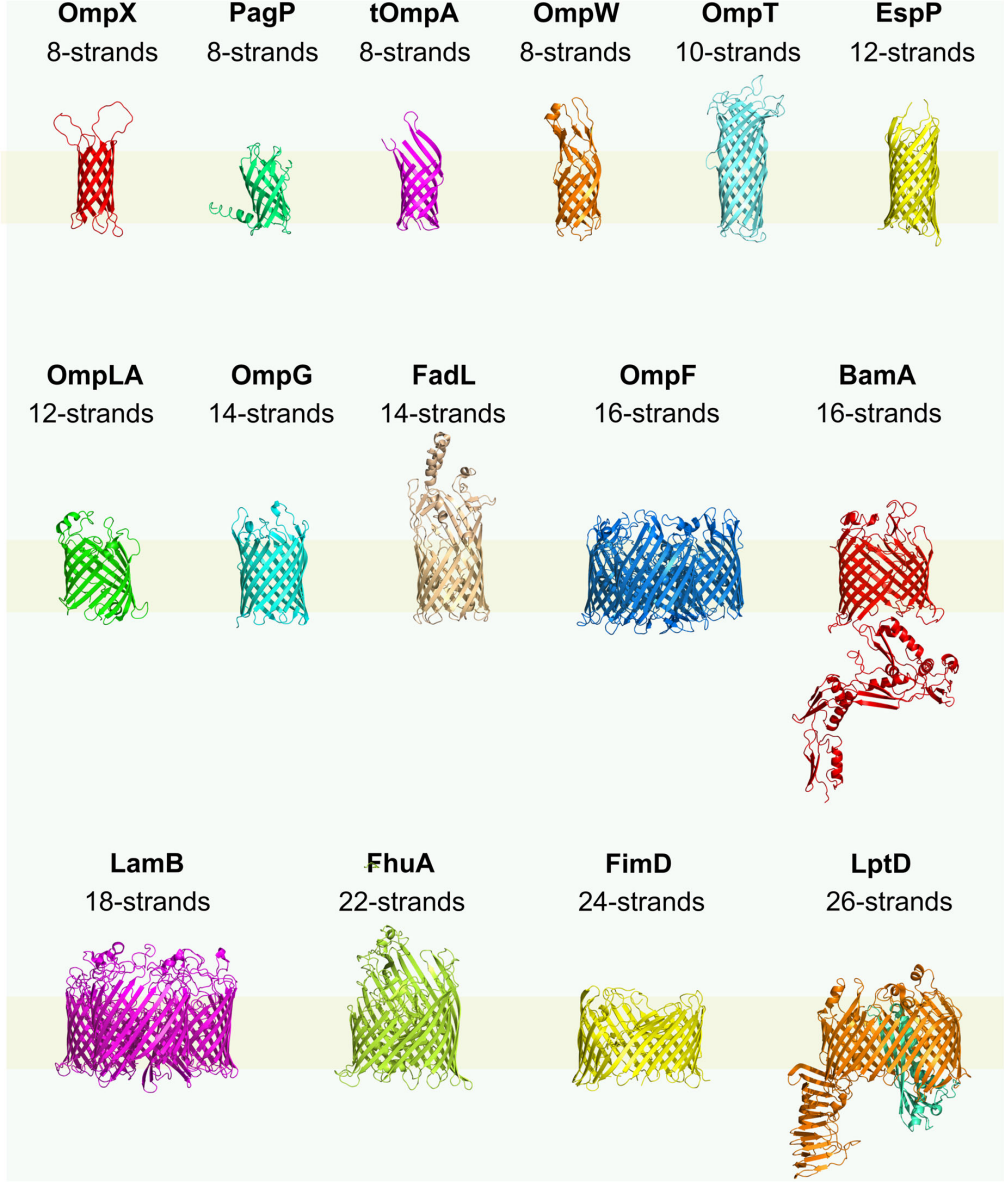
Crystal structures of OMPs from Gram-negative bacteria. *Top row*: OmpX (2MO6) [222]; PagP (3GP6) [73]; tOmpA (transmembrane domain of OmpA; 1QJP) [223]; OmpW (2F1V) [75]; OmpT (1 L78) [224]; EspP (β-domain) (2QOM) [225]. *Middle row*: OmpLA (1QD5) [226]; OmpG (2IWV) [227]; FadL (1T1L) [228]; OmpF (1OPF) [229]; BamA (4K3B) [76]. *Bottom row:* LamB (1MAL) [230]; FhuA (1BY3) [231]; FimD (3OHN) [232]; LptD (4Q35) [233]. All structures are from *E. coli* with the exceptions of BamA (*Neisseria gonorrhoeae*) and LptDE (*Shigella flexneri*). The LptD barrel has an associated lipoprotein subunit (LptE) in the functional complex (see main text). LptD and LptE are shown in orange and cyan, respectively. Approximate location of the membrane is shown in *yellow*, with the periplasmic face to the lower side of each structure. Note that OmpF and LamB are shown in their native trimeric forms

**Fig. 2:**
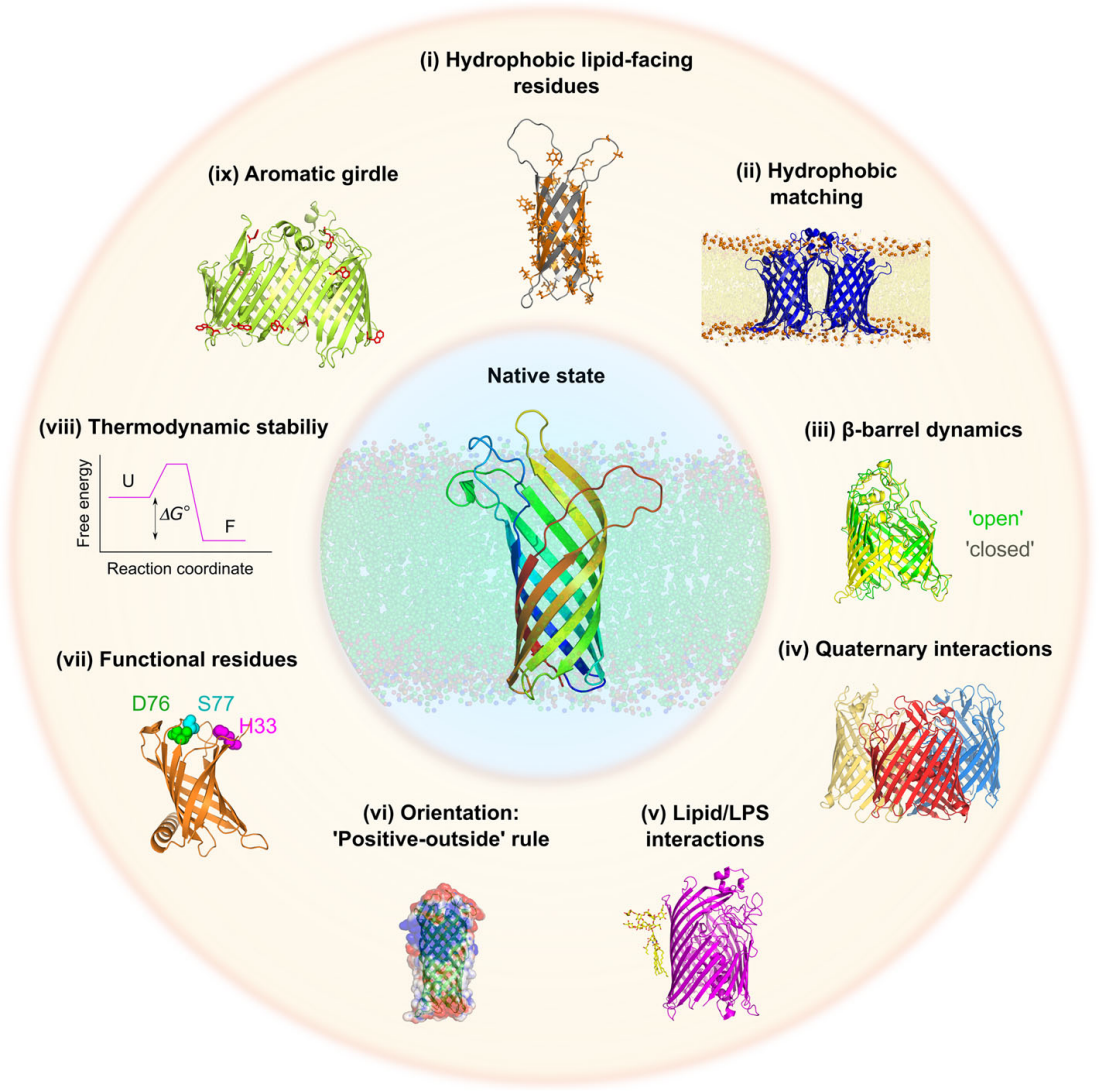
Requirements for OMPs to fold to a stable and functional state. Clockwise from *top*: (i) OmpX (2MO6 [222], *grey*)—hydrophobic residues are shown as *orange sticks*; (ii) OmpLA (1QD6 [226], *blue*) in a dimyristoylphosphatidylethanolamine (DMPE; *di*C_14:0_PE) bilayer (from [234]); (iii) alignment of BamA β-barrel structures in the ‘lateral open’ (5D0Q [78], *green*) and ‘lateral closed’ (5D0O [78], *yellow*) states; (iv) OmpF (2OMF [229])—monomers in the trimeric structure are shown in *red*, *yellow* and *blue*; (v) FhuA (1FI1 [235], *pink*)—bound LPS is shown as *yellow sticks*; (vi) OmpT (1I78 [224], *green cartoon*)—regions of *red* and *blue* represent areas of electronegative and electropositive surface potential (−1 kT/e to +1 kT/e) and were created using the APBS plugin for PyMOL [236]; (vii) PagP (3GP6 [237], *orange*)—conserved residues important in enzymatic function, H33, D76 and S77 (*pink*, *green* and *cyan*, respectively), are highlighted; (viii) free energy diagram showing the difference in stability of the folded (*F*) and unfolded (*U*) states; (ix) LptD (4N4R [37], *lime green*)—Trp residues are shown as *red sticks*. The central image shows the transmembrane domain of OmpA (1QJP [223], with mutated residues in the structure replaced with wild-type residues and missing residues in the loops built in using MODELLER [238]) in a DMPE bilayer (taken from [234])

Despite their sometimes complex topology, OMPs are able to fold spontaneously in vitro from their denatured states in urea or guanidinium chloride into detergent micelles or lipid bilayers [29–33] in the absence of cellular factors. This observation is consistent with Anfinsen’s findings for the water soluble protein ribonuclease A that all the information for folding is contained within the amino acid sequence [34]. OMPs are structurally and functionally diverse [35, 36], with those of known structure containing 8–26 membrane-embedded β-strands in their native state [35, 37] (Fig. 1). Larger β-barrel proteins can be formed by the assembly of monomeric subunits, such as CsgG (9 subunits, 36 β-strands) [38, 39] and GspD (15 subunits, at least 60 β-strands) [40, 41]. The OM of Gram-negative organisms is an asymmetric bilayer consisting of inner and outer leaflets formed from phospholipid and lipopolysaccharide (LPS), respectively, densely packed with proteins (protein:phospholipid:LPS ratio of 5:1:1 (*w*/*w*) [42, 43]). The requirement for OMPs to fold into this crowded membrane leads to significant pressures on the evolution of OMP sequences. These constraints can be placed broadly into two categories: (i) sequence requirements for OMPs to fold to a stable and functional native state in the OM (Fig. 2); and (ii) sequence requirements for assisted OMP assembly in vivo (see below). A major constraint is the need for solvation of hydrophobic amino acid side chains in the membrane; hence, the lipid-facing residues in OMPs must be overwhelmingly hydrophobic (Fig. 2(i)) [44], providing the drive for spontaneous membrane insertion [45]. This is reminiscent of the manner by which the hydrophobic effect provides an entropic drive towards the folding of water soluble proteins [46, 47]. Mutations which decrease the hydrophobicity of lipid-facing residues can reduce the kinetics of OMP folding in vivo, leading to premature degradation by the periplasmic protease DegP [48]. Interestingly, the eight-stranded OmpA is able to tolerate substitution of approximately two-thirds of its lipid-facing residues to other hydrophobic residues without losing its ability to fold or to be assembled by in vivo cellular machinery [49].

Recent breakthroughs have been made in measuring the thermodynamic stabilities of OMPs, with data now available for the eight-stranded proteins OmpA [50–54], PagP [55–57] and OmpW [56] and the 12-stranded OmpLA [58] (Fig. 1). These experiments have shown that OMPs are highly stable, with ΔG°_UN_ values ranging from −3.4 to −32.5 kcal/mol [16, 50, 56], consistent with their common resistance to denaturation by SDS [59–61]. Available evidence suggests that ΔG°_UN_ is correlated with the water-to-bilayer transition free energy of the residues that are in contact with the bilayer [56], calculated using the Moon-Fleming hydrophobicity scale [62]. The high stability of OMPs in the OM, compared with their lower free energy of binding to periplasmic chaperones (−7 to −13 kcal/mol [56, 63–65]), may serve as a sorting mechanism for OMPs in the periplasm [56], with the free energy of folding providing the driving force for assembly into the OM (note that the periplasm lacks ATP) [66, 67]. So, by contrast with water soluble proteins, which when folded are often only marginally stable [68], the balance between stability and function is less important for OMPs because of their high thermodynamic stability and high energetic barriers to unfolding [69, 70]. Such features should aid the evolution of new functionality since destabilising mutations that enhance a new function can be readily tolerated [71]. An interesting example of the trade-off between activity and stability has been suggested for the acyltransferase PagP: PagP from *Salmonella typhimurium* is twofold less stable than that from *Escherichia coli* in dodecylphosphocholine (DPC) micelles (−6.5 kcal/mol and −10.5 kcal/mol, respectively), but has 15–20-fold greater catalytic activity [72]. Interesting examples also exist where the stability gained by completing the hydrogen bonding between β-strands in the β-barrel has been sacrificed for functional requirements. This is the case for the β-barrels of PagP [73], FadL [74] and OmpW [75], whose structures suggest they may undergo opening movements to allow lateral entrance of substrates. Structures of BamA, the major subunit of the BAM complex (see below), revealed incomplete hydrogen bonding between the N- and C-terminal β-strands (β1 and β16) [76–79]. This results in frustration in the native protein, enhanced dynamics in the frustrated residues and increased ruggedness in the folding energy landscape close to the native state [80], the significance of which is not yet fully understood.

OMPs must also tailor their sequences to the local chemical characteristics of the OM. This results in different frequencies of residues at different membrane depths [81]. In particular, the positioning of aromatic residues in native OMP structures shows a strong preference for the membrane–water interface (‘aromatic girdle’) (Fig. 2(ix)) [81, 82], where they contribute to OMP stability [32, 57, 83]. Charged residues are also favoured at the chemically complex interfacial regions [81], with OMPs obeying a positive-outside rule (i.e. positively charged residues are located predominantly on the extracellular surface of the outer membrane; Fig. 2(vi)) [84], in contrast to the ‘positive-inside’ rule observed for α-helical MPs (i.e. basic residues are enriched on the cytoplasmic side of the membrane) [85]. This patterning of charges may help ensure correct orientation of OMPs in the OM. Burial of different residues in the bilayer makes different contributions to OMP stability, and recent evidence suggests that the free energy of partitioning of side chains into membranes is also dependent on the β-barrel scaffold [62, 86]. Computational analysis of the transfer free energies of residues at different membrane depths suggests that OMP orientation within the bilayer is also influenced by the effect of residue position on stability in the asymmetric OM [45]. Consistent with this finding, specific LPS binding sites have been identified for *E. coli* FhuA (Fig. 2(v)) [87], OprH from *Pseudomonas aeruginosa* [88] and *E. coli* OmpF, with the latter being shown to be important for biogenesis [89]. Lipid–protein interactions are also important for hydrophobic matching (Fig. 2(ii)). Indeed, the average hydrophobic thickness of OMPs, measured by the distance between aromatic girdles in OMP structures (23.7 ± 1.3 Å) [90], closely mirrors that of the OM in simulations [91]. The requirement for specific interactions between neighbouring β-strands also constrains OMP sequences. Glycine–aromatic inter-strand pairings are often found between neighbouring β-strands in water soluble proteins [92], and are also common in native OMP structures [84]. These may be important for OMP stability [84, 92] and folding [93], as well as having possible functional roles, as shown for autotransporter assembly [94]. Similarly to water soluble proteins, specific residues may be conserved since they form stabilising quaternary interactions (Fig. 2(iv)), or are required for enzymatic activity (Fig. 2(vii)) or barrel dynamics (Fig. 2(iii)) [95].

## Energy landscapes of OMP folding in vitro

**Fig. 3:**
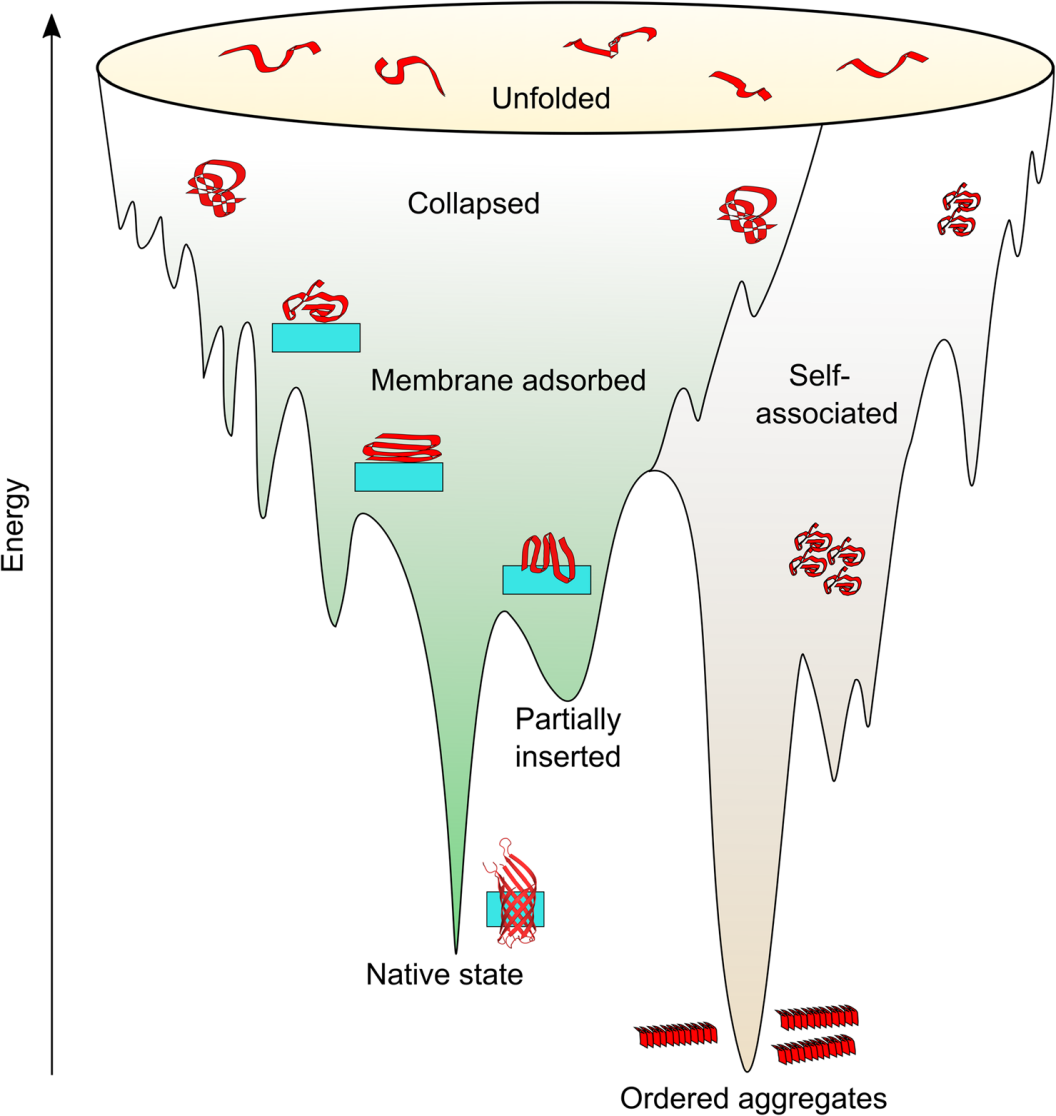
Hypothetical energy landscape for unassisted OMP folding. The *green surface* on the *left* depicts OMP conformations that may be formed en route to the native state. Non-native intramolecular interactions, and/or interactions with the membrane, may lead to ruggedness in the landscape. *The surface on the right* shows some possible conformations of self-associated OMPs, which may lead to the formation of ordered amyloid-like or disordered aggregates. How folding factors in the cell influence this landscape remains an open question. The OMP polypeptide chain and the membrane are shown in *red* and *blue*, respectively

Since the 1980s, experiments, simulations and theory have led to the view that funnel-shaped energy landscapes best represent the mechanisms of protein folding (Fig. 3), with the depth and width of wells in the landscape corresponding to the energy and conformational entropy, respectively [96–98]. The tension between the covalent connection between amino acid residues and the drive to minimise the contact free energy between each atom leads to frustration in the landscape [99]. Frustrated regions in proteins lead to increased ruggedness in the folding energy landscape, and hence kinetically trapped intermediate states become populated. Over 25 years of work on the folding of OMPs from denaturants into lipid membranes or detergent micelles in vitro has shown that OMPs fold on complex, rugged energy landscapes [29, 30, 100–102]. Early work on OmpA folding in vitro suggested a sequential pathway involving rapid formation of a collapsed state, followed by membrane absorption and possible partial membrane insertion of β-hairpins [103, 104]. OMP folding may, however, be more complex than a simple sequential route. For example, parallel folding pathways have also been proposed for OmpA [53, 105], FomA [69, 106] and PagP [107], dependent on the folding conditions, such as the pH, the nature of the lipid/detergent and the lipid:protein ratio. Recent, global analysis of the folding kinetics of tOmpA (the transmembrane domain of OmpA) into bilayers of different thicknesses, monitored by circular dichroism (CD) and SDS-PAGE, suggested a sequential model, with detours to off-pathway, misfolded states [108] (named a ‘predetermined pathway with optional errors’ [109]). This study revealed intermediates with a β-sheet content higher than that of the native state, which the authors propose may be due to transient β-strand formation by the extracellular loops on passage across the membrane [108]. Consistent with this view, recent in vivo experiments have shown that the assembly of BamA requires an extracellular loop (L6) to be buried within the newly forming β-barrel domain [110]. A model in which hydrophilic loops are tucked into folding OMP barrels to assist them across the hydrophobic membrane could also explain how lipids are prevented from entering the barrel lumen [110].

Available experimental evidence suggests a concerted mechanism for OMP folding in vitro, in which the final folding step and membrane insertion occur concurrently. Fluorescence quenching experiments showed that all four β-hairpins of the OmpA barrel cross the bilayer simultaneously [111], and similar kinetics were observed for the formation of OmpA secondary and tertiary structure (observed by CD and cold SDS-PAGE, respectively) on folding into *di*C_12:0_PC (DLPC) liposomes [112]. Consistent with this, at least partial structure is formed in all eight β-strands of PagP in its folding transition state [55]. Further, hydrogen-deuterium exchange (HDX) experiments, which monitored OmpX folding into detergent micelles, found that the rate of hydrogen bond formation was the same between all β-strands and synchronised with tertiary structure formation [113].

OMPs can also self-associate in their unfolded aqueous states [114], and can populate folded and unfolded dimers and trimers during folding experiments [115], as well as species with higher apparent molecular weights [108], adding further complexity to experiments tracking OMP folding mechanisms. The requirement for OMP sequences to contain alternating polar and non-polar residues that have a high propensity to form ordered aggregates [116] favours the formation of off-pathway, misfolded states. Indeed, similarly to the α-helical MP LacY [117], OmpA has been shown to form amyloid-like fibres in vitro in the absence of chaperones [118]. As fibrillar protein species can be associated with cellular toxicity [119, 120], this highlights the importance of chaperones in preventing aggregate formation in OMP folding (Fig. 3).

In vitro studies have highlighted the importance of the membrane environment on the kinetics of folding and insertion of OMPs [101, 121]. Bilayers that are more fluid, thinner, contain more unsaturated chains, and have increased curvature stress enhance OMP folding rates and yields [30, 33, 112, 121–124]. Bilayer properties also affect OMP thermodynamic stability; one study found that increasing curvature stress, by substituting C_16:0_C_18:1_PC (POPC) lipids for C_16:0_C_18:1_PE (POPE) in liposomes formed from POPC containing a 7.5% mole fraction of C_16:0_C_18:1_PG (POPG), increased the stability of OmpA. Conversely, substitution of POPC for shorter chain PC lipids (such as *di*C_10:0_PC) decreased stability [50]. In vitro studies have also begun to explore the influence of chaperones [105, 125, 126] and BAM protein components [127–132] on OMP folding in vitro. One key result from these studies is that when *E. coli* polar native lipids are used to create liposomes, there is a requirement for cellular folding factors to assist OMP folding [33, 127, 128], rationalising the conservation of these folding factors across bacterial species [133–135].

## OMP assembly in vivo

### Role of periplasmic chaperones

Unlike spontaneous folding that is often observed in very dilute solutions [34], protein folding in the cell is challenged by the high concentration of other proteins and macromolecules with which aberrant interactions can be made [136]. This can lead to aggregation, loss of function, and/or the accumulation of toxic species and cell death [119, 137]. Thus, cells expend considerable effort to maintain unfolded and partially folded proteins in a folding-competent state, and to degrade misfolded, aggregation-prone species [9, 10]. In the case of OMPs, folding is even more complex since the site of synthesis (the cytosol) is distal to the location of the final folded state in the OM [36]. A network of folding factors is thus required to ensure successful OMP folding and insertion into the OM, which becomes particularly important under stress conditions [138, 139].

**Fig. 4:**
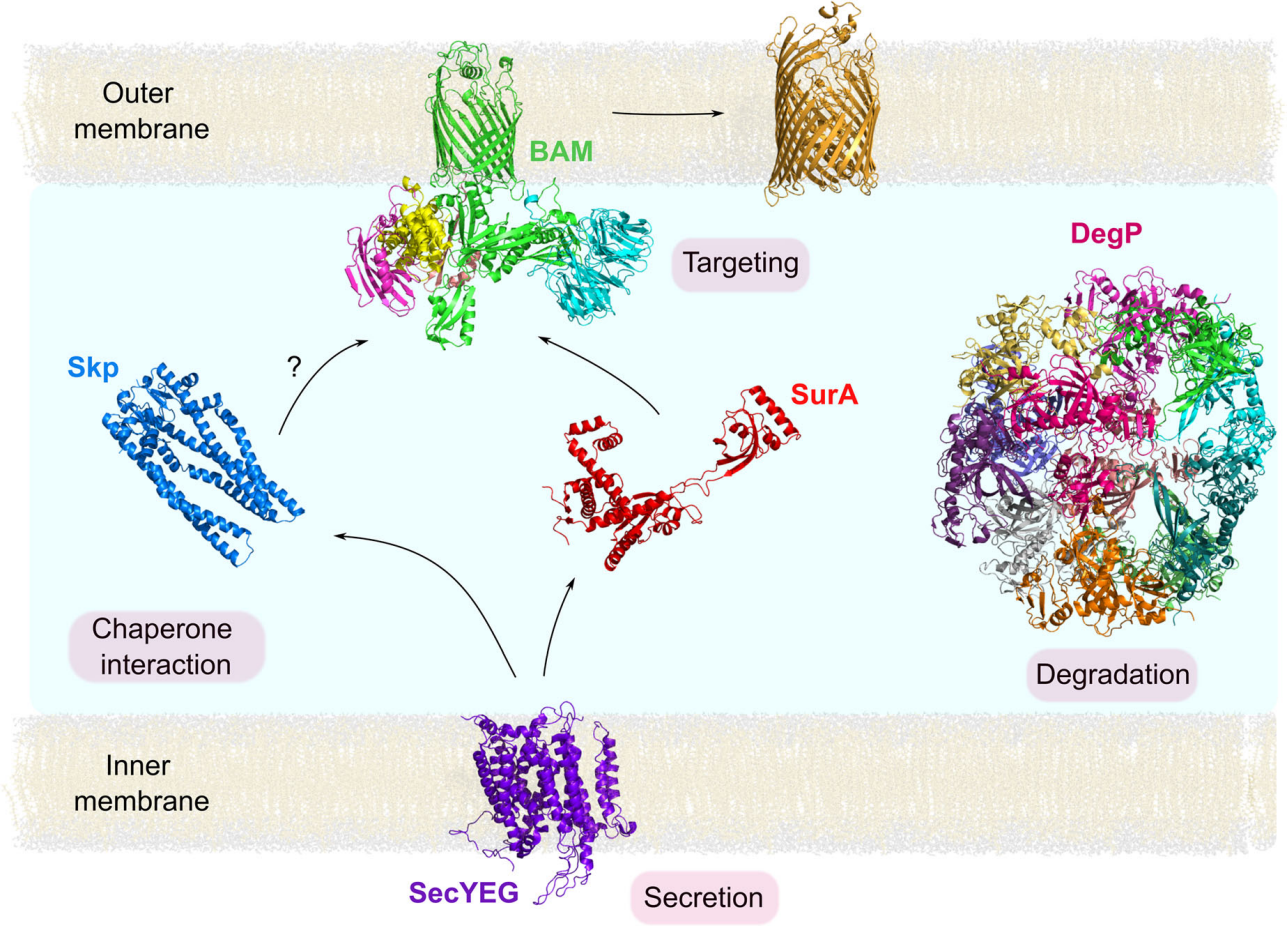
OMP assembly pathway across the periplasm. OMPs are secreted into the periplasm by SecYEG (*purple*, 5ABB [239]), where they are recognised by chaperones, of which the most important in *E. coli* are SurA (*red*, 1M5Y [148], with missing residues built using MODELLER [238]), and Skp (*blue*, 1U2M [240], missing residues built using PyMOL [241]). OMP sequences must also contain information for targeting to the BAM complex (5LJ0 [79]), which catalyses the final folding and insertion step into the OM. Misfolded OMPs trigger stress responses (e.g. DegS/RseA pathway [242, 243] (not shown)), and are degraded by the periplasmic protease DegP (shown with multiple colours highlighting the subunits of this dodecameric complex (2ZLE [244])). An example of a folded OMP in the OM (FhuA, 1BY3 [231]) is shown in *orange*. Note that LPS and the peptidoglycan layer are omitted from this schematic. The inner and outer membranes are shown approximated to the width of a di-oleoylphosphatidylethanolamine (*di*C_18:1_PE, DOPE) bilayer (generated in VMD [245]). The distance between the inner and the outer membranes can be inferred from the structures of machineries that span the periplasm, giving estimates in the range ~ 165–170 Å [179, 246] to ~ 190–210 Å [247]. Here, the latter distance (210 Å) is used, consistent with the dimensions of the periplasm observed by cryo-electron microscopy [248]. The *question mark* highlights the fact that whether Skp can deliver OMPs to BAM in vivo remains unknown, although BamA-containing membranes can promote folding of OMPs from their complexes with Skp in vitro [65, 126]

Following secretion into the periplasm via the SecYEG translocon in the bacterial inner membrane, OMPs are bound by chaperones, of which the major players in *E. coli* are SurA and Skp [138, 140]. While Skp is a homotrimer with an expandable hydrophobic cavity [141–144], SurA is monomeric and lacks an obvious protein binding site (Fig. 4). The kinetic competition between aberrant OMP self-association and chaperone binding is likely assisted by fast OMP–chaperone association rate constants (k_on_ ~ 1–3 × 10^8^ M^−1^ s^−1^) [64, 145] and by the availability of a reservoir of unbound chaperones [145]. SurA and Skp help to prevent OMP aggregation [64, 141], but whether they simply stabilise intermediate states on the folding pathway or are more actively involved in dynamically altering the energy landscape of folding to aid productive folding remains unresolved. NMR investigations of Skp-OMP complexes indicate that OMPs are held in an unfolded, compact, and highly dynamic state by multiple weak and transient interactions with Skp that contribute to its high avidity [146, 147], with binding affinities between Skp and OMPs in the low nanomolar range [56, 63]. This high entropy-low enthalpy chaperone-bound state is consistent with the notion that the unusually high thermodynamic stability of OMPs acts as a free energy sink, providing the driving force for release of OMPs from their chaperone-bound states and their folding into the OM [56, 67].

Less is known about the conformations of OMPs bound to SurA. As the structure of this chaperone lacks a cage-like cavity (Fig. 4) [148], clients may bind SurA in a more extended conformation, as observed for substrates binding to the chaperone trigger factor [149] and SecB [150], both of which bind OMPs in the cytosol [28]. Similarly to Skp [143], multiple copies of SurA may bind to different regions of unfolded OMPs simultaneously, possibly in a ‘beads-on-a-string’ fashion [65, 151]. Such extended OMP conformations could prevent the formation of collapsed states, facilitating subsequent folding, as well as disfavouring the formation of aggregates. Consistent with this, mechanical unfolding experiments have shown that SurA can stabilise FhuA folding intermediates and promote their refolding into a bilayer via sequential β-hairpin units [151]. By contrast, in similar experiments Skp prevented misfolding, but did not assist refolding [151], suggestive of a significant difference in the mode of action of these two chaperones.

SurA is known to have a preference to bind Ar-X-Ar and Ar-Ar motifs (where Ar = aromatic and X = any amino acid) that are commonly found in OMP β-strands [152, 153]. How these interactions bias the conformational ensemble towards on-pathway folding intermediates remains unresolved. Interestingly, Skp and SurA can bind a broad repertoire of substrates, including a wide range of OMPs [154, 155], as well as water soluble proteins [156–159], including the model protein Im7 in both its native and unfolded states [160]. Unfolded states of OMPs in denaturant have been shown to exhibit non-random coil behaviour [161–163], raising the possibility that OMP–chaperone binding may be entropically favoured by increasing the conformational freedom of the OMP substrate upon chaperone binding [163].

Soluble domains attached to an OMP β-barrel can also provide chaperone-like behaviour for their attached β-barrel domains [164]. Such chaperoning may also occur in larger barrels that contain plug domains, such as the 22-stranded Ton-dependent transporters (e.g. FhuA (Fig. 1) [165]) and the 26-stranded LptD, which has a separate protein, LptE, residing within the native LptD barrel (Fig. 1) [37]. By providing a surface for nascent β-sheets to form around, domains within large barrels may also assist OMP folding by preventing misfolding and smoothing the energy landscape for folding [166].

When folding fails misfolded OMPs are recognised, triggering stress responses such as the σ^E^ response (reviewed elsewhere [139, 167–170]). This leads to upregulation of chaperones, and the chaperone/protease DegP, which degrades OMPs in the periplasm (Fig. 4) [140]. In addition, the proteases BepA [171] and YcaL [172] are able to degrade misfolded OMPs that have already engaged with the BAM complex. How OMPs that have successfully folded into the OM are turned over was not well understood since *E. coli* lacks both ATP and a ubiquitin-protease system in the periplasm. Recent exciting experiments using fluorescence microscopy in vivo showed that turnover of OMPs is achieved during cell division, wherein old OMPs are moved to the poles and passed to a fraction of daughter cells following binary fission [173, 174]. Akin to the chaperone network in the cytosol, therefore, in which the concentration of chaperones, foldases and proteases is carefully balanced to sustain life, even under stressful conditions [175], OMP synthesis, folding and degradation are also finely balanced in the bacterial periplasm. Indeed, recent kinetic simulations of OMP biogenesis showed that the flux of OMPs across the periplasm can be modelled as a stochastic process, controlled by the thermodynamics and kinetics of OMP interactions with folding factors and their concentrations [145].

### Insertion and folding into the OM

The headgroups and acyl chain lengths of lipids in the OM provide a significant kinetic barrier for OMP folding [33, 65, 127]. Nature has answered this problem by the creation of an ATP-independent catalyst (the BAM complex) that catalyses folding and insertion of OMPs into the OM (Fig. 4). In *E. coli*, BAM is a ~ 203 kDa heteropentameric complex (BamA–E) [28, 176–184]. Two BAM subunits are essential: the evolutionarily conserved BamA (itself an OMP; Fig. 1) [185, 186] and the lipoprotein BamD [187]. Deletion of BamB, BamC or BamE, by contrast, leads to varying degrees of *E. coli* growth defects [176].

Nascent OMPs must be recognised by BAM and released from their chaperones before they can fold into the OM. The mechanisms of chaperone release are currently not well understood, but must occur in the absence of ATP, contrasting markedly with the mechanism of action of cytosolic chaperones and chaperonins [9, 10]. SurA has been found in OM fractions [153] and has been cross-linked to BAM in vivo [188, 189]. By contrast, evidence for a direct interaction between Skp and BAM is lacking [188], although BamA-containing proteoliposomes promote the folding of Skp-bound OmpA and tOmpA in vitro [65, 126]. Alternatively, Skp may use electrostatic interactions between its positively charged surface and negatively charged lipid headgroups in the inner leaflet of the OM (phosphatidylglycerol (PG) and cardiolipin [190]) to assist delivery of OMPs directly to the OM [105, 125]. A conserved sequence at or near the C-termini of OMPs (known as the β-signal) is important for efficient OMP assembly [191, 192]. It has been suggested that this sequence is involved in targeting OMPs to BAM via interactions with BamD [193, 194] and/or BamA [195], although structural evidence for such a molecular recognition event is still lacking.

**Fig. 5:**
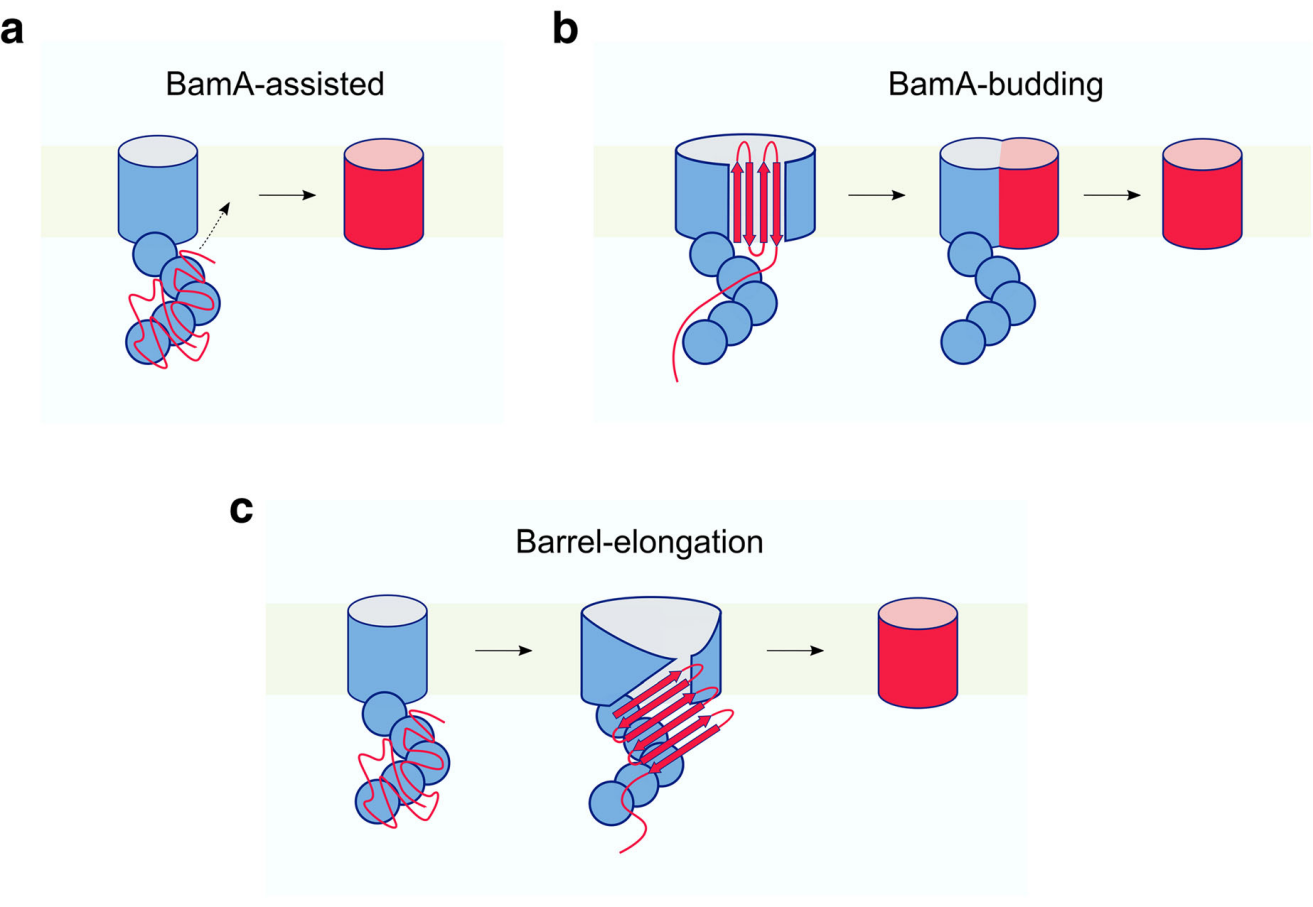
Possible, currently hypothetical, mechanisms for OMP assembly by the BAM complex. **a** BamA-assisted. OMPs may fold via a pathway similar to that observed in vitro*,* with BamA acting as a membrane ‘disruptase’ to assist folding. **b** BamA-budding. OMP assembly involves the formation of a hybrid barrel by sequential insertion of β-strands templated by the β1/β16 strands of BamA. When the final substrate β-strand has been inserted, the nascent OMP buds off from the BamA barrel to complete folding. **c** Barrel-elongation. Interaction of the nascent OMP with the periplasmic BAM region promotes a ‘lateral open’ BAM state, exposing the β1 strand of BamA. BamA β1 then templates β-sheet formation in the nascent OMP, possibly via β-hairpin units. Folding is completed by concerted OMP insertion and tertiary structure formation, releasing the BamA barrel and allowing BAM to return to the ground state. In all models BamA is involved in destabilising the membrane to aid insertion and folding (not shown). The lipoproteins BamB–E and the chaperone SurA have been omitted for clarity. Note that there is currently little direct experimental evidence to favour one model over another (see main text for more details)

How BAM catalyses OMP folding remains a second open question. The presence of BAM in the OM may smooth the OMP folding energy landscape by destabilising trapped intermediates and/or lowering the activation energy of folding. Alternatively, BAM may accelerate folding by changing the structural mechanism of OMP folding such that large energy barriers are avoided. Several models have been proposed for the mechanism of BAM-catalysed OMP folding, including the widely publicised ‘BamA-assisted’ and ‘BamA-budding’ models [178, 182] (Fig. 5a, b). The BamA-assisted model proposes that BAM reduces the kinetic barrier to folding by thinning and disordering lipids close to the BamA barrel seam, which facilitates OMP folding and insertion (Fig. 5a). In support of this model, the hydrophobic thickness of BamA is reduced at the β1–β16 seam, and molecular dynamics simulations of lipid-embedded BamA showed local membrane thinning and disordering at this region [76]. The creation of membrane defects by maintaining lipids at their transition temperature has also been shown to accelerate OMP folding [196]. Further, BamA alone has been shown to increase OMP folding kinetics in vitro [65, 126, 127, 132], with BamA acting as a more effective catalyst in thicker bilayers [65].

In the BamA-budding model, a sequential pathway is proposed for β-strand insertion, in addition to membrane priming (Fig. 5b). In this model, the C-terminal β-hairpin of nascent OMPs is proposed to be threaded into the BamA barrel lumen, triggering lateral opening of the BamA barrel at the β1–β16 seam. These unpaired β-strands of BamA are then envisaged to interact with the nascent OMP via β-augmentation to form an OMP–BamA hybrid barrel. Sequential addition of further OMP β-strands to this ‘super-barrel’ occurs until the last strand of the OMP is added, triggering budding of the OMP and restoration of BamA to the closed state for a new folding cycle [178, 197]. A similar hybrid barrel model has been proposed for the assembly of autotransporters by the BamA homologue TamA [198]. There is no direct evidence for the BamA-budding model [181], although cross-linking of the BamA β1–β16 seam has been shown to be lethal in vivo [80, 197]. However, cross-linking could simply reduce the kinetics of assembly such that cells are no longer viable [182], consistent with the finding that cross-linking the BamA β1–β16 seam impairs, but does not prevent, BAM-mediated folding of OmpT in vitro [79].

An alternative model proposed for BAM function suggested tetramerisation of BamA to create a pore that facilitates OMP folding and insertion into the OM [176]. Recently determined BAM structures suggest that such a model is highly unlikely given the potential steric clashes between periplasmic BAM components in the hypothetical tetrameric BAM assembly [77–79, 199]. The finding that a single copy of BAM in nanodiscs containing *E. coli* polar lipid extract is able to assemble the autotransporter EspP also argues against this model [131]. Spatial clustering of BAM complexes may be functionally relevant, however, as BAM has been observed in 0.5 μm ‘OMP islands’ in vivo [173], and genetic experiments suggest that multiple copies of BAM may be involved in the assembly of trimeric porins [200].

**Fig. 6:**
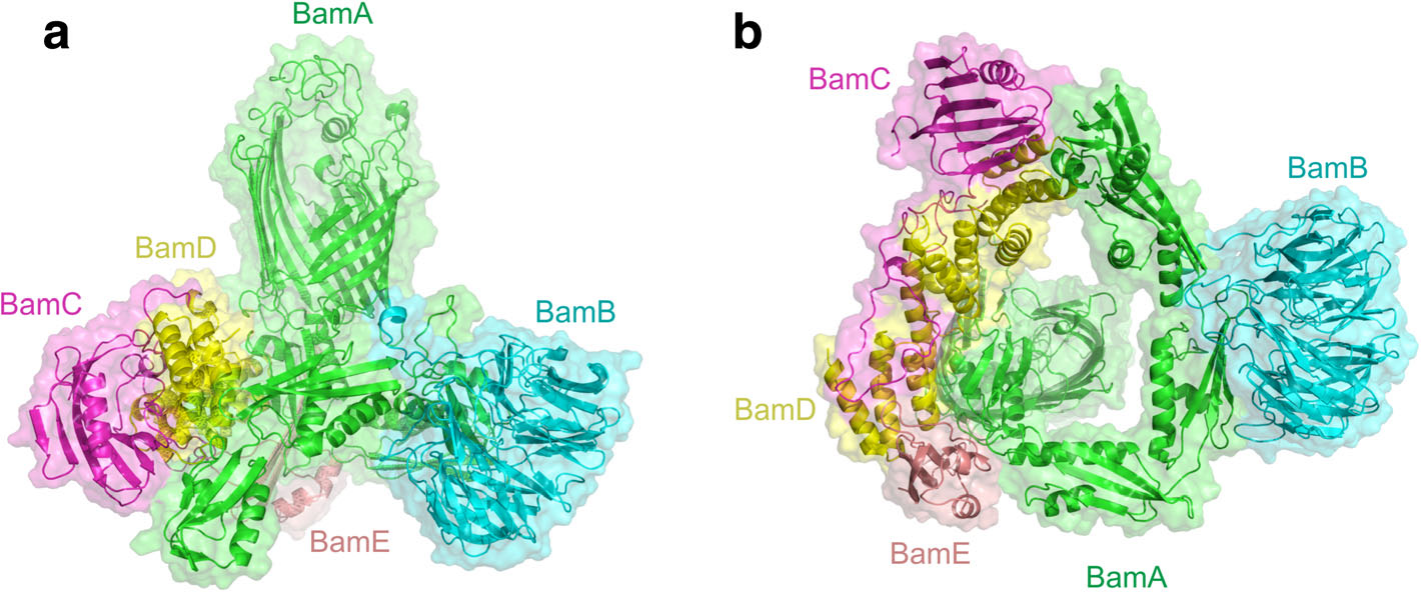
Cryo-EM structure of the BAM complex. Solution structure of the BAM complex viewed from **a** the membrane plane, and **b** the periplasm. Image created with PyMOL (5LJO [79]). Individual BAM subunits are labelled in different colours, as indicated

Here, we propose an alternative ‘barrel-elongation’ model for BAM action (Fig. 5c). In this model, a lateral-open state of BamA within the BAM complex is considered the OMP-acceptor, with catalysis of OMP folding involving β-strand augmentation by the β1 strand of BamA. This templating mechanism is analogous to the elongation phase of amyloid self-assembly reactions in which β-strands are added sequentially to the growing end of amyloid fibrils [119, 201]. In addition, non-specific aggregation is minimised by folding in a protected environment created by the POTRA domains and BamB–E (see below; Fig. 6a, b). Hence, energetic barriers to folding are proposed to be lowered by templated association of neighbouring β-strands, consistent with in vitro studies that have shown this is the rate-limiting step of unassisted OmpA folding into liposomes [202]. In the proposed model, the formation of OMP β-sheet structure is hypothesised to begin in the periplasm, in agreement with recent in vivo cross-linking data for an LptDE complex stalled on BAM, which indicated a partially folded LptD barrel in the periplasm [166]. It is possible that successive β-strands are added to nascent OMPs in β-hairpin units. This would be consistent with the observations that bacterial OMPs: (1) have short turns at their periplasmic sides, often consisting of only a couple of residues, in contrast with the characteristically long loops on their extracellular sides [35, 203]; (2) have both N- and C-termini at the periplasmic side of the OM; and (3) all have an even number of β-strands (Fig. 1) [203]. Further, AFM mechanical unfolding experiments have shown that OMPs can unfold [204, 205] and refold [151] via β-hairpin units. Whether or not OMP assembly occurs via association of preformed β-hairpins, however, remains to be seen.

Recent publications reporting structures of BAM in different conformational states have shown that BamB–E, together with the N-terminal polypeptide transport-associated (POTRA) domains of BamA, form a ring-like structure in the periplasm (Fig. 6a, b) [77–79, 199]. Delivery of OMPs by SurA to this region may trigger opening of the BamA barrel to initiate OMP folding. Interactions of nascent OMPs with BamD may be important in this process [206, 207], and dynamics between POTRAs 2 and 3 may also be key to the formation of this active state [208–210]. The BAM periplasmic ring may also provide a cage-like environment, which could be extended by the binding of SurA, to protect elongating OMPs from aberrant interactions in a manner analogous to the ‘folding cage’ of chaperonins [9, 10]. The barrel-elongation model, therefore, proposes that the BAM catalytic effect is achieved by: (1) ordered, sequential release of the OMP polypeptide chain from chaperones into the periplasmic BAM folding funnel; (2) catalysis of β-structure formation in the periplasm by a β-augmentation interaction with β1 of the BamA barrel; and (3) membrane disruption to facilitate concerted insertion and OMP tertiary structure formation. Not all of these catalytic features may be required for the folding of every OMP. For example, templated β-sheet formation in the nascent OMP by β1 of the BamA barrel may not be essential for the assembly of smaller OMPs. While experimental evidence for the barrel-elongation model is lacking, it is consistent with evidence from genetic studies of the BAM complex [206, 207, 211, 212], and with current knowledge of autotransporter assembly [131, 213–218], which is dependent on the BAM complex [131, 216]. Importantly, a peptide from the OMP FimD (Fig. 1) was recently cross-linked to the TamA barrel, consistent with an interaction with TamA β1 via β-augmentation [219]. Much more work will be needed to provide evidence for or against the different models for BAM activity proposed here (barrel-elongation) and elsewhere (e.g. BamA-assisted, BamA-budding) [176, 181, 182], and to determine whether different mechanisms are utilised for different substrates [200]. In particular, determination of a structure of an intermediate along the BAM-mediated OMP assembly pathway may enable different models to be ruled in or out. Such stalled intermediates were, for example, important in elucidating the mechanism of pilus assembly by the FimD usher [220]. Efforts to obtain the structure of a nascent OMP–BAM complex stalled during folding are a logical next step in the quest to elucidate how this fascinating molecular machine sculpts the OMP folding energy landscape, enabling efficient control of folding and membrane insertion in the absence of ATP.

## Towards realistic models of OMP folding energy landscapes

Great progress has been made in our understanding of how OMPs fold in vitro into bilayers formed from different lipids. Similarly, impressive breakthroughs have been made in understanding the factors required for OMP assembly in vivo. How the pathways of OMP folding in vitro compare with those in vivo, however, remains unclear, partly due to a lack of studies that use direct biophysical measurements of OMP folding into physiologically relevant membranes to complement powerful, but indirect, in vivo methods. In vivo, chaperones and the BAM insertase are required to sculpt the OMP folding energy landscape to ensure rapid and efficient folding into the crowded OM. Several models for the mechanism of BAM catalysis of OMP folding have been proposed. All involve membrane destabilisation close to the BamA barrel, with some implicating a more direct role of BamA itself in the formation and stabilisation of OMP folding intermediates. Recent progress in structural analyses of BAM in different conformational states, combined with the development of biochemical and biophysical methods able to track the progress of OMP folding [221], promise to cast new light on the mechanisms of OMP folding, and how OMPs are recognised by their chaperones and released for folding by BAM. Such knowledge will aid the task of bringing our understanding of the folding mechanisms of OMPs to the level of detail achieved for water soluble proteins. It will also allow realistic models of the energy landscapes of OMP folding to be drawn, including how the landscape is modulated by the asymmetric OM and the BAM complex. Combined with impressive achievements in genetic and cellular studies of OMP assembly, there is no doubt that exciting discoveries will be reported in the years ahead, fuelled by the incentive that such knowledge may lead to the generation of much-needed new anti-bacterial agents that target OMP biogenesis.
